# Determining the Location of the Fovea Centralis Via En-Face SLO and Cross-Sectional OCT Imaging in Patients Without Retinal Pathology

**DOI:** 10.1167/tvst.10.2.25

**Published:** 2021-02-17

**Authors:** Archana A. Nair, Rebecca Liebenthal, Shefali Sood, Grant L. Hom, Marc E. Ohlhausen, Thais F. Conti, Carolina C. S. Valentim, Hiroshi Ishikawa, Gadi Wollstein, Joel S. Schuman, Rishi P. Singh, Yasha S. Modi

**Affiliations:** 1 Department of Ophthalmology, NYU Langone Health, New York University, New York, NY, USA; 2 Center for Ophthalmic Bioinformatics, Cole Eye Institute, Cleveland Clinic, Cleveland, OH, USA; 3 Case Western Reserve University, Cleveland OH, USA

**Keywords:** fovea centralis, OCT, retina

## Abstract

**Purpose:**

The purpose was to establish the position of the fovea centralis to the optic nerve via en-face, near-infrared spectral domain optical coherence tomography (NIR-OCT) in healthy patients. This may shed light on physiological variability and be used for studying subtle cases of foveal ectopia in macular pathology and after retinal detachment.

**Methods:**

SD-OCT data of 890 healthy eyes were retrospectively analyzed. Exclusion criteria included axial myopia causing tilting of the optic disc, peripapillary atrophy >1/3 the width of the disc, macular images excluding greater than half of the optic disc, and patients unable to maintain vertical head positioning. Two independent reviewers measured the horizontal and vertical distance from the fovea to the optic disc center and optic disc diameter via cross-sectional and en-face scanning laser ophthalmoloscopy OCT imaging.

**Results:**

890 eyes were included in the study. The right and left eyes differed in the horizontal distance from the fovea to the disc center (4359 vs. 4248 µm, *P* < 0.001) and vertical distance from the fovea to the disc center (464 µm vs. 647, *P* < 0.001). This corresponded to a smaller angle between the right and left eyes (6.07° vs. 8.67°, *P* < 0.001). Older age was associated with a larger horizontal (*P* = 0.008) and vertical distance (0.025). These differences persisted after correcting for axial length in the 487 patients with axial-length data.

**Conclusions:**

This study compares the position of the fovea centralis among individuals without macular pathology on a micron level basis. The significant variability between right and left eyes indicates that contralateral eye evaluation cannot be reliably used. Rather, true foveal ectopia requires assessments of preoperative and postoperative NIR-OCT scans. This finding is relevant to retinal detachment cases and evaluation of subtle foveal ectopia.

**Translational Relevance:**

This finding is relevant to retinal detachment cases and evaluation of subtle foveal ectopia.

## Introduction

Outcomes in retinal detachment surgery have tremendously improved. Modern studies report anatomic success after the first procedure in more than 90% of individuals.^1^^–^^4^ However, a significant minority of patients with macula-off retinal detachments report persistent visual symptoms including dysmetropsia, distortion of image size,^5^ or metamorphopsia, distortion of image form.^6^^,^^7^ These symptoms are believed to be related to retinal slippage and micron-to-millimeter–level transposition of the retina.^8^ Fundus autofluorescence highlights large-scale inferior retinal slippage, often with postoperative “ghost vessels.” These hyper-autofluorescent “ghost vessels” are thought to be areas of retinal pigmented epithelium, previously located under the blood vessels but exposed to light postoperatively. Comparing the “retinal pigmented epithelium ghost vessel” to the retinal vasculature provides information on the degree of retinal displacement.^9^ In fact, foveal ectopia or retinal displacement after surgery may be seen in up to 60% to 72% of patients.^8^^–^^11^ Although this imaging modality can identify large retinal transpositions, it is not ideal for identifying micron-level slippage. Often, patients may be symptomatic despite normal FAF imaging, and thus a search to identify micron-level changes in retinal transposition may be valuable.

**Figure 1.**
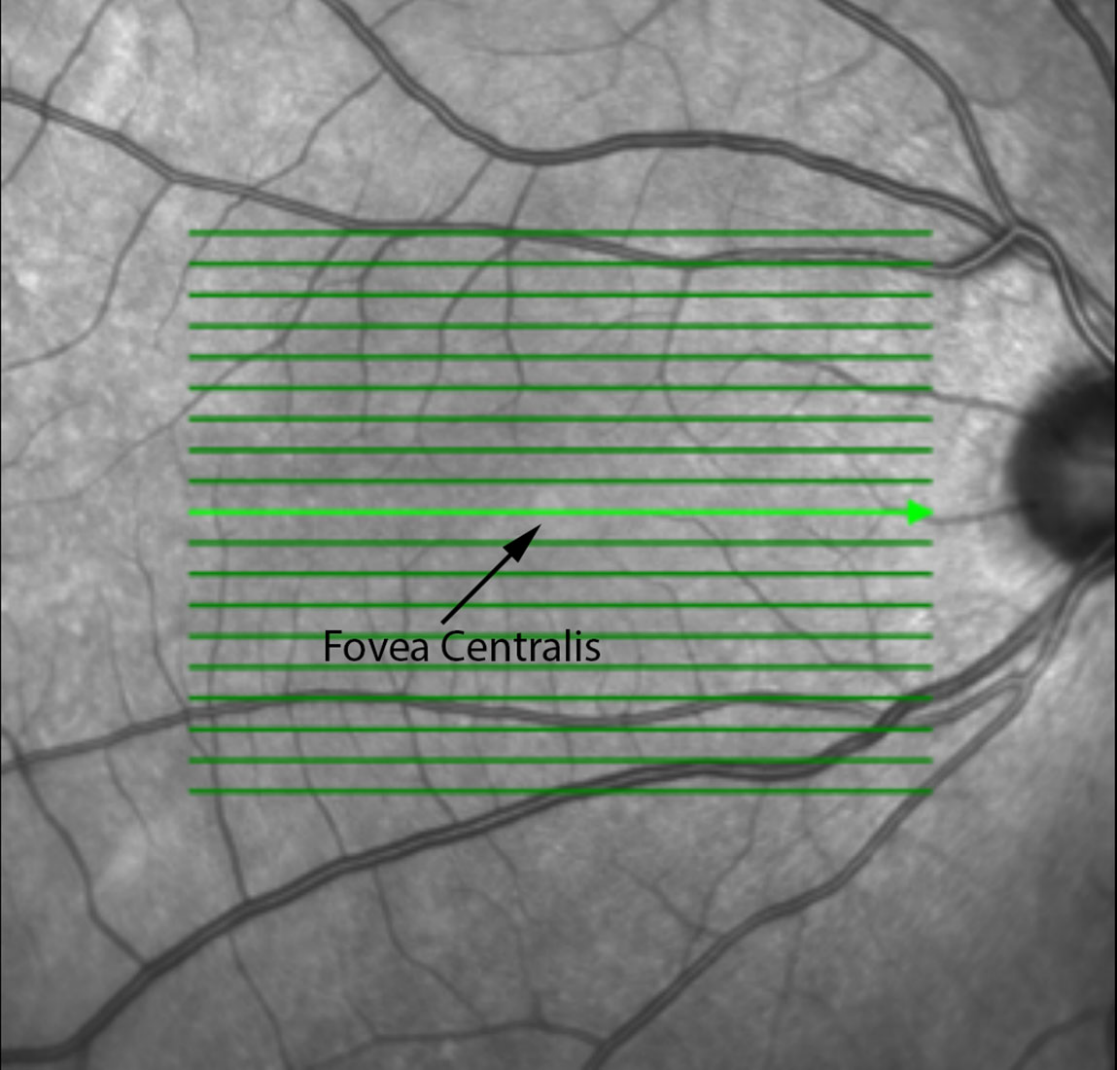
En-face SLO OCT with fovea centralis and optic disc.

One possible imaging modality to assess this with greater accuracy is the near-infrared scanning laser ophthalmoscopy (SLO) image on optical coherence tomography (OCT), which highlights the fovea centralis with ease (Fig. 1). However, it is unknown where the “true” position of the fovea centralis is on OCT and whether this changes with age, gender, axial length, or between eyes.

The purpose of this study was to establish the position of the fovea centralis using both cross sectional and en-face SLO OCT imaging in a normative database of healthy patients of varying ages. Knowledge of the normative position of the fovea centralis on OCT may inform physiological variations and potentially, shed light on symptomatic patients after retinal detachment.

## Methods

This study was a retrospective cross-sectional review of patients seen at two institutions: New York University (NYU) and the Center for Ophthalmic Bioinformatics at the Cole Eye Institute, Cleveland Clinic. The study was approved by the Institutional Review Board with a waiver of consent at both NYU and Cleveland Clinic. The research adhered to the tenants of the Declaration of Helsinki and complied with the Health and Insurance Portability and Accountability Act of 1996.

### Patient Selection

Patients without macular disease or strabismus who underwent spectral domain optical coherence tomography (SD-OCT) who came for general ocular examinations were included in the study. The protocol was to image patients with their head vertical. Special attention was made to ensure vertical alignment of the forehead and chin to normalize the torsional corrective moments of the eye before scanning. Patients were excluded from the study if they had (a) axial myopia causing tilting of the optic disc, (b) peripapillary atrophy greater than 1/3 the width of the disc, or (c) OCT imaging excluding greater than half of the optic disc. Additionally, patients with glaucomatous cupping, retinopathy, torticollis, and kyphosis were excluded.

### Measurements and Data Collection

**Figure 2.**
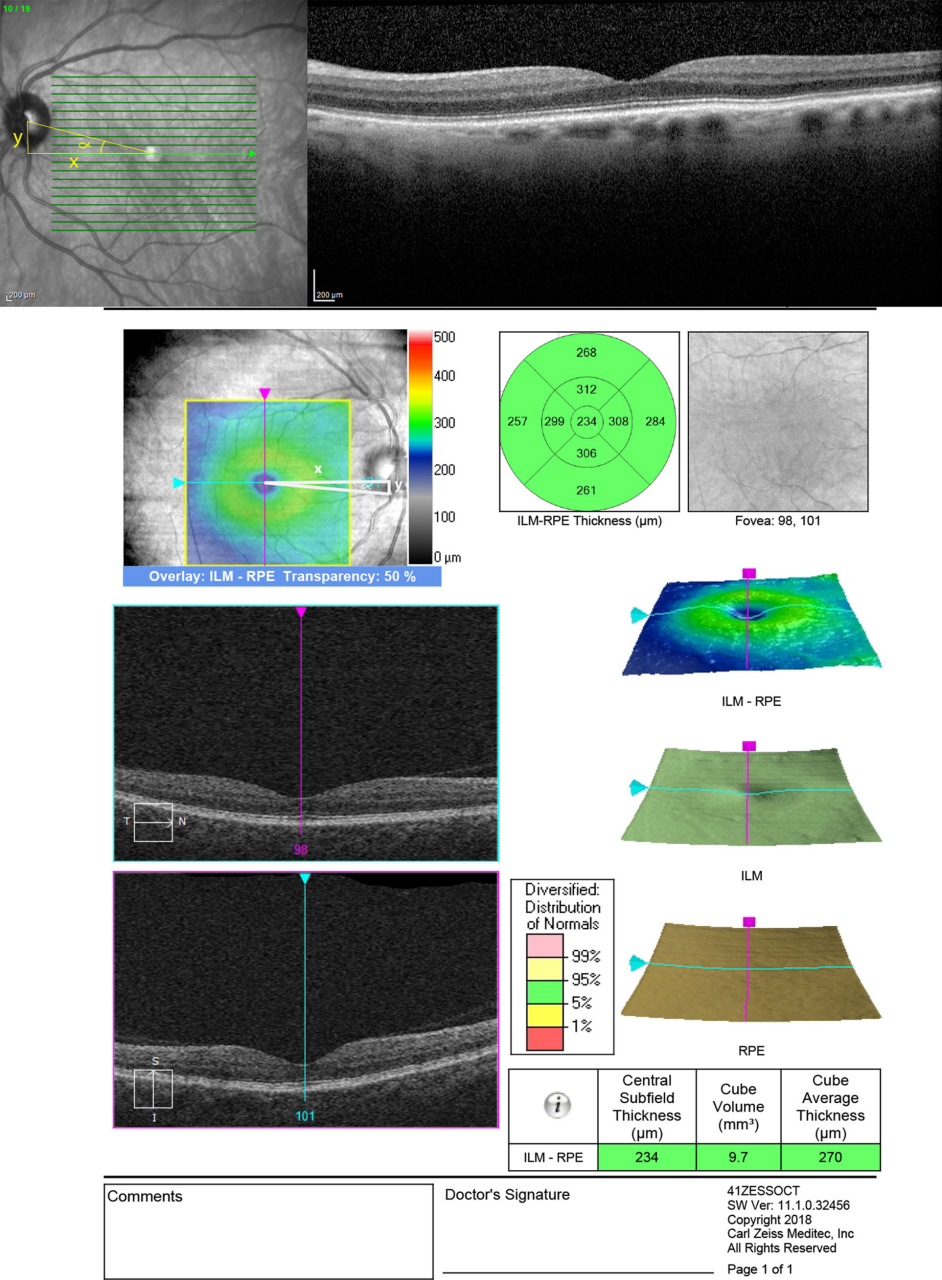
En-face SLO SD-OCT imaging. X measures the horizontal distance from the fovea centralis to the optic disc center. Y measures the vertical distance from the fovea centralis to optic disc center. Alpha represents the angle between the fovea centralis and the optic disc center.

SD-OCT measurements were conducted by two masked readers (A.N. and R.L) at NYU and (M.O and G.H.) at the Cole Eye Institute. Imaging was conducted on Spectralis Heidelberg (Heidelberg Engineering, Heidelberg, Germany) and Cirrus (Zeiss, Dublin, CA, USA) OCT platform. Technical specifications of the Spectralis include a scan size of 496 pixels, axial image resolution 3.9 µm/pixel, lateral resolution of 5.7 µm/pixel. Specifications for the Cirrus image include an axial image resolution 5 µm and lateral resolution 10 µm/pixel. For each en-face SLO OCT image of the macula, the following measurements were conducted: (a) the horizontal distance from the foveal center to the optic disc center (ODC, X on Fig. 2), (b) the vertical distance from the foveal center to the inferior disc margin (Y on Fig. 2), and (c) vertical disc height (Fig. 2) on the OCT machines using in-built software. All patients were only imaged on one imaging platform. The apex of the superior and inferior nerves was identified, and a vertical line connecting the two was made to identify the vertical disc height. The foveal center was identified on the near-infrared imaging by a hyperreflective spot. This area was cross-referenced with the foveal dip on the cross-sectional OCT to ensure that the hyperreflective foveal centralis seen on SLO matched the true anatomic foveal dip. Repeat measurements were done for patients with multiple imaging studies at different time points to evaluate for consistency of measurements at different scanning time points. The angle alpha was calculated from the data. Demographic information including age and gender along with refraction, when available, were obtained via chart review. Axial length was collected, when available, with chart review. If refraction data were unavailable and patients’ uncorrected visual acuity was 20/20 on Snellen testing, refraction was estimated as plano.

### Statistical Analysis

For each patient, the variability between the right and left eyes for each parameter was compared. The inter observer agreement was calculated using a Cohen's kappa score. A cutoff of κ > 0.61, or substantial agreement, was used. If the kappa statistic was found to be less than the cut off, discrepant measurements were made by a third independent reviewer and the values most in agreement were used. Measurements with greater than a 750-µm difference between users were also reviewed by a third independent reader. Continuous dependent variables were compared using a paired *t*-test, continuous independent variables were compared using a *t*-test, and categorical variables were compared using χ^2^ analysis. A linear regression was used to further compare the variables. A Littmann correction to correct for the ocular magnification in OCT was conducted where axial length measurements were available. The following formula was used:

$$\begin{array}{c} t=p\times q\times s. \end{array}$$

The *t* represents the corrected value, *p* is the magnification factor of the machine, *q* = 0.01306 × (axial length measured – 1.82), and *s* equals the measurement obtained on OCT. A *P* value of 3.382 was used for measurements obtained on Cirrus, and 3.393 was used for measurements obtained on the Spectralis Heidelberg.^12^ Statistical significance was defined as *P* < 0.05. All statistical analyses were conducted using Stata 14.2 (StataCorp, College Station, TX, USA).

## Results

In total, 444 patients (888 eyes) were included in the study. One hundred twenty patients were imaged on the Spectralis Heidelberg, all done at NYU, and 325 were imaged on the Cirrus (Zeiss) OCT platform, 84 at NYU, and 240 at Cole Eye. 277 patients were female (62.4%) and the average age of the study population was 62.5 years (Table 1). Spherical equivalent of eyes ranged from −6 D to +5.25 D. Axial length data was available for 487 (54.8%) eyes. Axial length ranged from 20.34 mm to 27.65 mm (mean 23.88, SD 1.13).

**Table 1.** Demographic Information

	Heidelberg (n = 218)	Cirrus (n = 650)	*P* Value
Age (years)	50.49	67.0	<0.001
Age group (%)			<0.001
<30 years	34 (15.60)	14 (2.15)	
30–39 years	32 (14.68)	12 (1.85)	
40–49 years	36 (16.51)	30 (4.62)	
50–59 years	50 (22.94)	84 (12.92)	
60–69 years	36 (16.51)	210 (32.31)	
>70 years	30 (13.76)	300 (46.15)	

**Figure 3.**
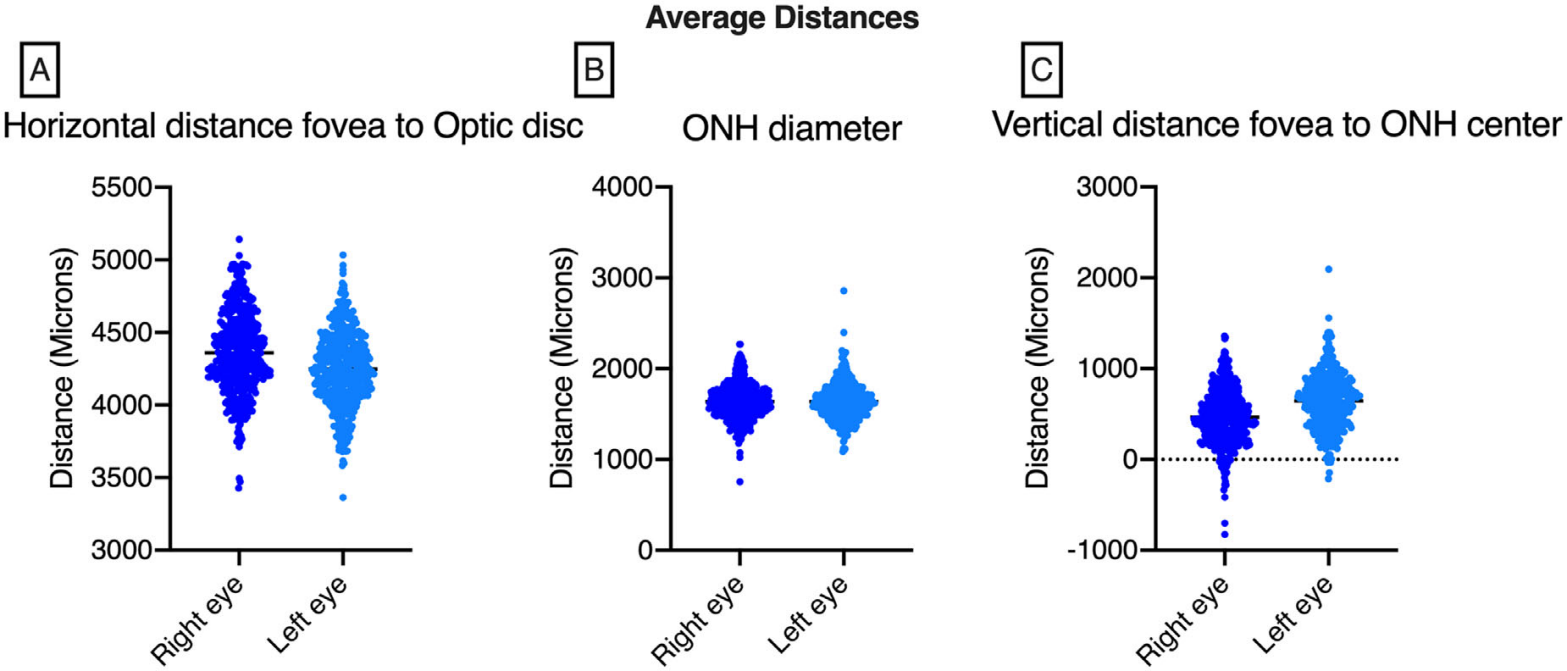
(A) Average horizontal distance of the fovea to optic disc comparing right versus left eye. (B) Average optic nerve head diameter of the right versus left eye. (C) Average vertical distance of the fovea to the optic disc right versus left eye. (D) Interuser variability horizontal distance of the fovea to the optic disc comparing right versus left eye. (E) Interuser variability of the optic nerve head diameter comparing right versus left eye. (F) Interuser variability of the vertical distance of the fovea to the optic disc right versus left eye.

A small but statistically significant difference was found between the right and left eyes in the horizontal distance from the fovea to the disc center (4359 vs. 4248 µm, *P* < 0.001, Table 2) and remained statistically significant after conducting a Littman correction accounting for axial length (4312 vs. 4173, *P* < 0.001, Table 3). This difference was much more pronounced, however, when assessing the vertical distance from the fovea to the disc center (464 µm vs. 647 µm in the right and left eyes, respectively; *P* < 0.001, Table 2) and persisted after accounting for axial length (481 vs. 617 µm in the right and left eyes, respectively; *P* < 0.001, Table 3). Correspondingly, a statistically significant difference in angle (α) was found between the right and left eyes (6.07° vs. 8.67°, *P* < 0.001, Table 2) and remained significant after correction (6.07° vs. 8.68 °, *P* < 0.001, Table 3) No statistical difference was seen in the optic disc diameter between the right and left eyes (1640 vs. 1639 µm, *P* = 0.950). Figure 3 plots the position of the right and left eye measurements for optic nerve head diameter, horizontal distance from fovea to optic nerve center, and vertical distance from fovea to optic nerve center. No statistically significant interobserver difference was seen for each of the three measurements (Table 2).

**Table 2.** En-face SLO OCT Measurements of the Right and Left Eyes, Averaged From the Two Independent Reviewers

	Right Eye	Left Eye	*P* Value	Interuser Agreement (Kappa Statistic)	Agreement Over Multiple Scans (Kappa)
Horizontal distance from fovea to disc center (microns)	4359	4248	<0.001	0.79%	0.913
ONH diameter (microns)	1640	1639	0.950	0.90%	0.973
Vertical distance from fovea to disc center (microns)	464	647	<0.001	0.68%	0.955
Angle	6.07°	8.67°	<0.001		

ONH, optic nerve head.

**Table 3.** En-face SLO OCT Fundus Measurements of the Right and Left Eyes, Corrected for the Axial Lengths, Averaged from the Two Independent Reviewers

	Right Eye	Left Eye	*P* Value
Horizontal distance from fovea to disc center (microns)	4312	4173	<0.001
ONH diameter (microns)	1609	1595	0.430
Vertical distance from fovea to disc center (microns)	481	617	<0.001
Angle (degrees)	6.07°	8.68°	<0.001

A total of 487 eyes had axial length data available.

ONH, optic nerve head.

**Figure 4.**
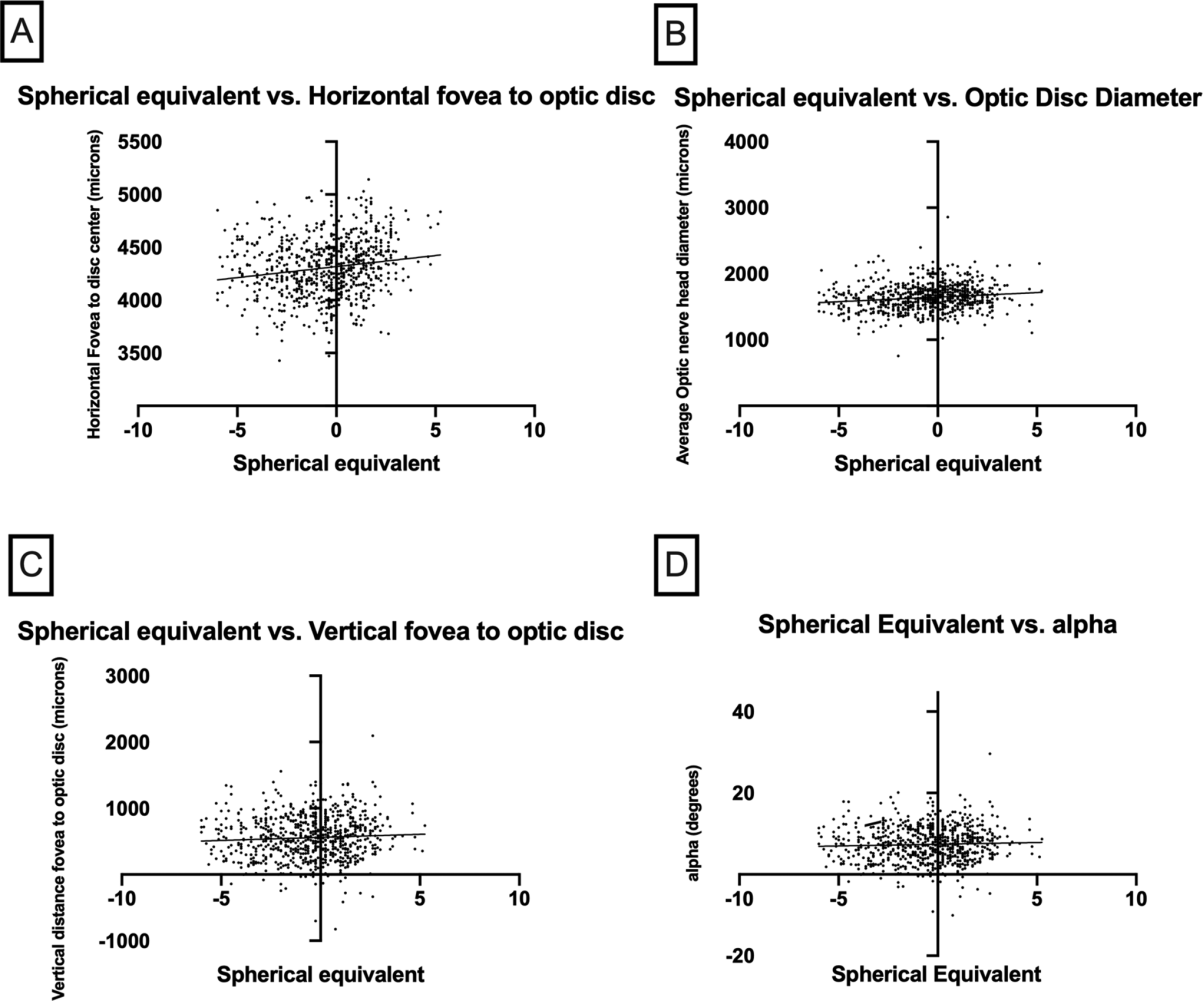
(A) Average horizontal distance of the fovea to optic disc versus the spherical equivalent. (B) Average optic disc diameter versus spherical equivalent. (C) Average vertical distance of the fovea to optic disc versus spherical equivalent. (D) Angle alpha versus spherical equivalent. The *lines* represent the linear regression of each dataset and highlights a correlation between hyperoptic refraction and increased horizontal distance.

To assess reproducibility between measurements, a small cohort of 20 eyes with repeat imaging on different days was assessed. The average number of repeat scans analyzed was 3. The change in X and Y axis measurements was 3.3 (range 0–388, *P* = 0.91) µm and 20.7 µm (range 0.25 – 245, *P* = 0.32), respectively. There were no statistically different measurements detected, demonstrating repeatability of the measurements across multiple patient visits (Table 2). The difference in time points was not found to make a statistically significant difference on the measurements. The range of time points for repeat measurements was 0 to 38 months. A multivariate analysis adjusting for age and gender found no statistically significant difference between the right and left eyes for optic nerve head diameter (*P* = 0.371). Refraction was not available for 38 patients. A multivariate analysis adjusting for age, gender, laterality showed that more hyperopic eyes have larger horizontal distance from fovea to optic nerve head center (*P* < 0.001) and larger optic nerve head diameter (*P* < 0.001). These associations, while statistically significant, were both modest with significant splay of the data points (Fig. 4^5^). No statistically significant association was found between the spherical equivalent and vertical distance from the fovea to optic nerve head center (*P* = 0.349) or between the spherical equivalent and the angle between the fovea and optic nerve head center (*P* = 0.670).

**Figure 5.**
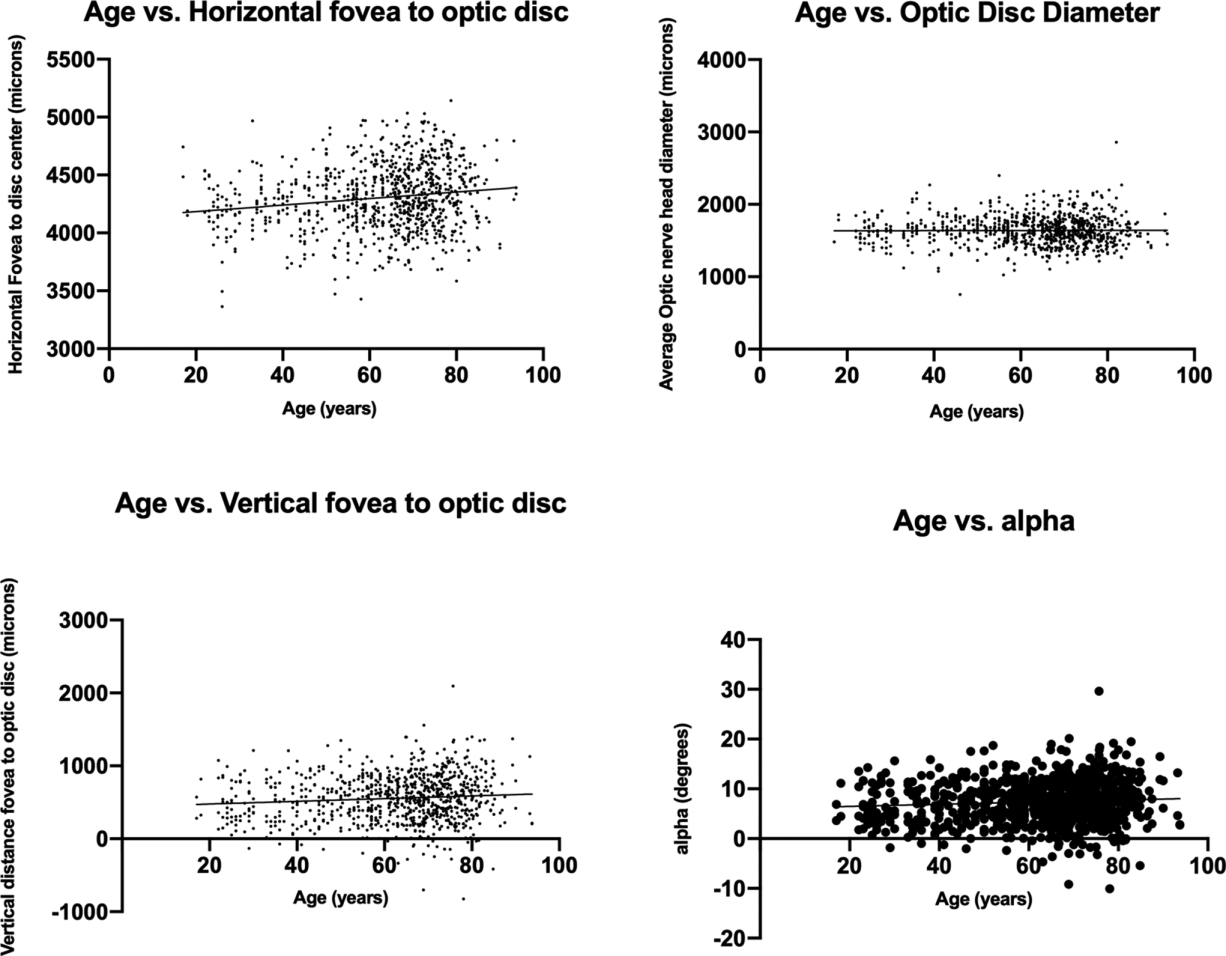
(A) Average horizontal distance of the fovea to optic disc versus age. (B) Average optic nerve head diameter versus age. (C) Average vertical distance of the fovea to optic disc versus age. (D) Angle alpha versus age. The line represents the linear regression of the dataset and demonstrates a modest correlation between increased age and increased horizontal distance.

There was a significant difference in the vertical distance from the foveal disc to optic nerve centers between males and females in multivariate analysis. The position of the fovea centralis was located on average 57 µm below the vertical position of the fovea centralis in men compared to women (*P* = 0.005), which persisted after accounting for axial length (*P* < 0.001). No statistically significant difference was found between men and women in horizontal distance or optic nerve head diameter. Older age was correlated with a larger vertical distance (*P* = 0.008); average vertical distance was 482 µm for patients younger than 30 years, 487 µm for patients between 30 to 40 years of age, 518 µm for patients between 40 to 50 years, 559 µm for patients between 50 to 60 years, 558 µm for patients between 60 to 70 years, and 581 µm for patients older than 70 years. Advanced age, however, was not correlated with horizontal distance of the fovea centralis (*P* = 0.347). Older age was also correlated with a larger horizontal distance (*P* = 0.025). Average horizontal distance was 4173 µm for patients younger than 30 years, 4280 µm for patients between 30 to 40 years, 4195 µm for patients between 40 to 50 years, 4292 µm for patients between 50 to 60 years, 4313 µm for patients between 60 to 70 years, and 4347 µm for patients older than 70 years.

There was a statistically significant difference between Cirrus and Spectralis in horizontal distance between fovea to optic nerve head center (4147 µm vs. 4357 µm, *P* < 0.001). There was no statistically significant difference in vertical distance (568 µm vs. 552 µm, *P* = 0.52) or optic nerve head diameter (1636 µm vs. 1645 µm, *P* = 0.582) (Fig. 5).

## Discussion

This study demonstrates a normative range of the position of the fovea centralis using the combined NIR en-face SLO OCT and cross-sectional OCT imaging. Some notable findings are apparent. First, the “normal” position of the fovea centralis, as measured by OCT, can vary dramatically from one patient to the next (vertical range −824.75 to −2093.5 µm) and between eyes (vertical range −834.5 to −1378.5). When applying this information to pathologic states, it thus becomes critical to assess each eye independently and compare the preoperative to the postoperative state to make true assessments on foveal ectopia. Although no preoperative and postoperative measurements were taken in any patient because this captured normal data, the high interuser and interscan agreement for the measurements highlights that this is a viable technique to consistently address the true position of the fovea centralis over different patient visits, particularly if the same imaging system is used across visits.

Prior studies have used various imaging modalities to evaluate the position of the fovea centralis.^13^^–^^17^ The methodologies are mostly similar with measurements of the center of the optic nerve to the fovea centralis recorded. However, there were variations in imaging modalities, with most studies using fundus photos^13^^,^^14^^,^^16^ and few studies using alternative imaging modalities.^17^^,^^18^ Most studies report similar angles of displacement (α), with the range of these studies being between −5.6° to 7.76°.^16^^,^^17^ However, there are notable differences. Although many studies did not compare right and left eyes, Rohrschneider^17^ and van de Put et al.^14^ noted that there were similarities in the vertical deviation of the foveal centralis between eyes. This finding, however, was not reproduced in this study. The vertical deviation between eyes may be a true anatomic variation between eyes versus a torsional deviation (excyclotorsion) in the left eye at the time of OCT image acquisition. Although vertical head position was scrutinized prior to image acquisition, torsional effects on the globe may still be at play and this may be an inherent limitation of OCT image acquisition when assessing the fovea centralis. The second hypothesis of torsional effects playing a role at the time of OCT acquisition, is favored given that other imaging modalities demonstrated similar vertical positions of the fovea centralis. This indicates that the position of the fovea centralis relative to the optic nerve head is also likely dependent on the imaging modality used, but reproducibly similar in the same patient across repeat OCT scans on the same imaging platform.

Increasing axial length has been previously reported to increase the distance from the optic nerve head center to the fovea centralis.^14^^,^^16^^,^^19^ This was not reproduced in this study as hyperopic eyes were associated with a modestly increased horizontal distance from the fovea to the optic nerve head and overall optic nerve head diameter. This difference, however, was small. Axial length imaging was present in just over half of patients and a Littman correlation was performed to account for this variability in ocular magnification differences in eyes with different axial lengths. Given that over half of eyes had this correction and measurements were similar across the subgroup with axial length measurements with Littman correlation corrections and the whole group, these changes cannot be entirely explained by lateral magnification effects inherent to OCT acquisition for different axial lengths.

In a study by Chihara et al.,^20^ increasing age was found to be associated with increased fovea to disc distance, independent of axial length. This was reproduced in our study with older patients having increased horizontal and vertical distance from fovea to disc center. This suggests that age is an independent modifier of the fovea to optic disc distance and may be related to the hyperopic shift seen in aging patients.^21^

A study by Giani et al.^18^ looked at the differences in spectral-domain OCTs to determine whether there were any inconsistences between devices. They concluded that there was a strong correlation between devices. Although the Spectralis and Cirrus OCTs may use different scan patterns, they did not find differences in retinal anatomic landmarks. Our study, however, showed no difference in vertical distance or disc diameter. However, horizontal distance demonstrated a small difference among the different imaging modalities. Thus it is likely important to ensure not only same eye comparisons, but also, same OCT platform scans.

There are some limitations of the study that are noteworthy. This was a retrospective cross-sectional analysis of the fovea centralis via SD-OCT SLO imaging across two platforms. Given the retrospective nature, data on axial length were not available for all patients. There is also some inherent variability in the measurements across time points. This is mainly due to the quality of the near-infrared images displayed on the OCT machines. Therefore a threshold value should be used to determine whether there are any clinically significant changes in pathological states. Unfortunately, few of our patients had repeat scans available given the retrospective nature of the study. Additionally, for this normative study, we excluded those with significant peripapillary atrophy (PPA) and thus this study was not designed to evaluate high myopes. A strict head positioning protocol was applied to patients in this study to ensure upright positioning and normalization across patients. This imaging modality, however, is unable to control for the extent of torsion which can alter the position of the fovea centralis. This may certainly have accounted for the difference between eyes.

Nonetheless, this study was conducted on two imaging platforms on a high number of patients, which adds to the strength of the conclusions. It also demonstrates reproducibility of measurements on the same individual at different times and provides meaningful information on the location and variability of the foveal position between individuals. Collectively, these data provide insights on the utility of OCT as a tool to evaluate micron-level changes in pathologic states. It highlights that same eye comparisons across the same platform are likely to provide the best assessment of true foveal ectopia. Further imaging modalities in these pathologic states is currently underway and are shaped by this normative, pilot study.
